# Preoperative management and anticoagulant efficacy in atrial myxoma-associated acute ischemic stroke: a case report and literature review

**DOI:** 10.3389/fcvm.2024.1435047

**Published:** 2024-11-12

**Authors:** Yu Chen, Qian Huang, Chengping Bai, Hao Zhang, Bo Zhang

**Affiliations:** ^1^ School of Clinical Medicine, Qinghai University, Xining, Qinghai, China; ^2^ Department of Neurology, Qinghai University Affiliated Hospital, Xining, Qinghai, China

**Keywords:** atrial myxoma, stroke, anticoagulant, clinical presentation, case report, literature review

## Abstract

Acute ischemic stroke (AIS) is a common complication of atrial myxoma (AM), and most emboli originate from a thrombus attached to the surface of the myxoma, with occasional shedding of tumor fragments leading to stroke. Clinical manifestations of AM include mitral valve obstruction, systemic embolism, and weakness. However, neurological deficits and other nonspecific manifestations may mask the presence of AM. The surgical resection is the most effective treatment for these conditions. However, the main problem is the lack of consensus regarding the prevention and treatment of stroke complicated by myxoma during the perioperative period. This study aims to improve the diagnosis and treatment of AM and the associated AIS. Here, we report the case of a 27-year-old patient with AM who presented with chest pain, palpitations, and sudden dizziness and had a stroke under anticoagulant treatment during the admission period. We also reviewed and summarized the clinical presentations and characteristics of similar previously reported cases. Our review emphasized the importance of early diagnosis and appropriate perioperative management of AM and its associated AIS.

## Introduction

Primary cardiac tumors are rare, with an incidence of 0.05%, and 90% are benign tumors, including 50% myxomas (1). Left atrial myxoma is a benign primary cardiac tumor with an annual incidence of approximately 0.5 per million (2), mostly affecting women aged 30–60 years (3).

Myxoma is often gelatinous, and its tumor surface is smooth or dented, with various clinical manifestations related to its morphology, mobility, fragility, and location (2). Typical triads of symptoms include embolization, mitral obstruction, weakness, and other symptoms such as vertigo, cough, fever, and even asymptomatic patients (4). The cerebral infarction mainly involved in the distribution area of the internal carotid artery (5). If a myxoma is not removed in time, there is a high risk of embolization and sudden cardiac death caused by mitral stenosis. However, due to its complex and atypical symptoms, the timely diagnosis of AM is very challenging.

Although the most effective treatment is surgical resection, little attention has been paid to the perioperative management of patients, and the efficacy of anticoagulation in preventing embolization resulting from AM remains unclear. In this study, we report the case of a 27-year-old patient with AM who presented with chest pain, palpitations, and sudden dizziness and had a stroke under anticoagulant treatment during the admission period. We also reviewed and summarized the clinical presentations and characteristics of similar previously reported cases to improve the diagnosis and treatment of AM and its associated AIS.

## Case report

A 27-year-old female patient was admitted to the hospital for “dizziness with visual rotation for 1 day”, accompanied by nausea, vomiting gastric content, abdominal pain, and diarrhea symptoms. She did not have any risk factors for cerebrovascular diseases such as hypertension, diabetes, dyslipidemia, atrial fibrillation, coronary heart disease, congenital heart disease, cardiomyopathy, arteritis, and other diseases. There was no history of an unclean diet, infection, smoking, alcohol consumption, or recent use of oral contraceptives. In the past 5 months, the patient experienced recurrent palpitations, chest pain, and fatigue. Chest pain was defined as persistent, crushing pain, which mostly appeared after activity and did not radiate to the shoulder or back. Each episode lasted for several minutes and was relieved after changing positions or resting. Extreme low-density imaging was observed in the left temporal region of the patien's head Computed tomography (CT) scan; however, after consultation, there was no history of stroke or neurological deficit, nor any family history of stroke, heart disease, or other genetic diseases. A heart examination revealed low heart sounds and no murmur in the cardiac auscultation areas. Pulmonary detection was normal, with no lower extremity edema or peripheral vascular features. Neurological examination revealed no ataxia or nystagmus, bilateral pupillary reflex examination was also good, and the remaining physical examinations showed no abnormalities. After admission, laboratory examinations indicated elevated inflammatory markers and cardiac biomarkers (Table 1). There were no other abnormal indicators, and the electrocardiogram indicated sinus bradycardia and paired premature ventricular beats.

**Table 1:** The patien's laboratory examination results.

Laboratory study	Result	Reference range
Erythrocyte sedimentation rate (mm/h)	47 mm/h	≤20 mm/h
C-reactive protein (mg/L)	4.82 mg/L	≤6 mg/L
Creatine kinase (U/L)	859 U/L	40–200 U/L
Creatine kinase isoenzyme (U/L)	38 U/L	<24 U/L
Myoglobin (ng/mL)	166 ng/mL	<70 ng/mL
Aspartate aminotransferase (U/L)	64 U/L	13–35 U/L

Given that the patient was a young female and the nature of the suspected intracranial lesion was unclear, we performed a cranial magnetic resonance imaging on the second day after admission, which confirmed the softening lesion in the left temporal lobe (Supplementary Figure S1). At that time, we considered unstable angina pectoris, and the patient was diagnosed with endocarditis and arrhythmia. However, the cause of the intracranial lesions is yet to be elucidated. The patient complained of intermittent fever and decreased endurance over the past few months. Considering that the patient may have had myocardial damage, diarrhea, and increased levels of inflammatory indicators, only symptomatic treatment was administered.

**Figure 1:**
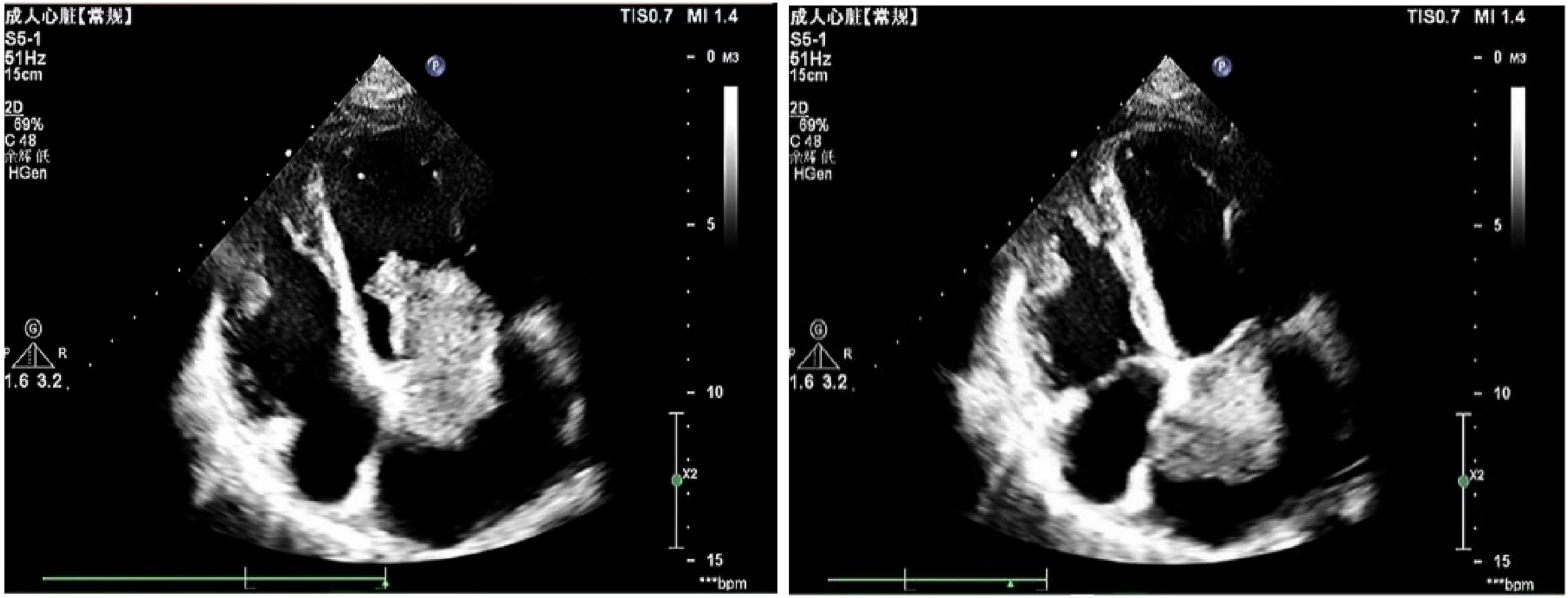
An irregular left atrial echogenic mass, about 32 mm × 34 mm, well defined and active, enters the left ventricle through the mitral valve orifice in diastole and enters the left atrium in systole.

**Figure 2:**
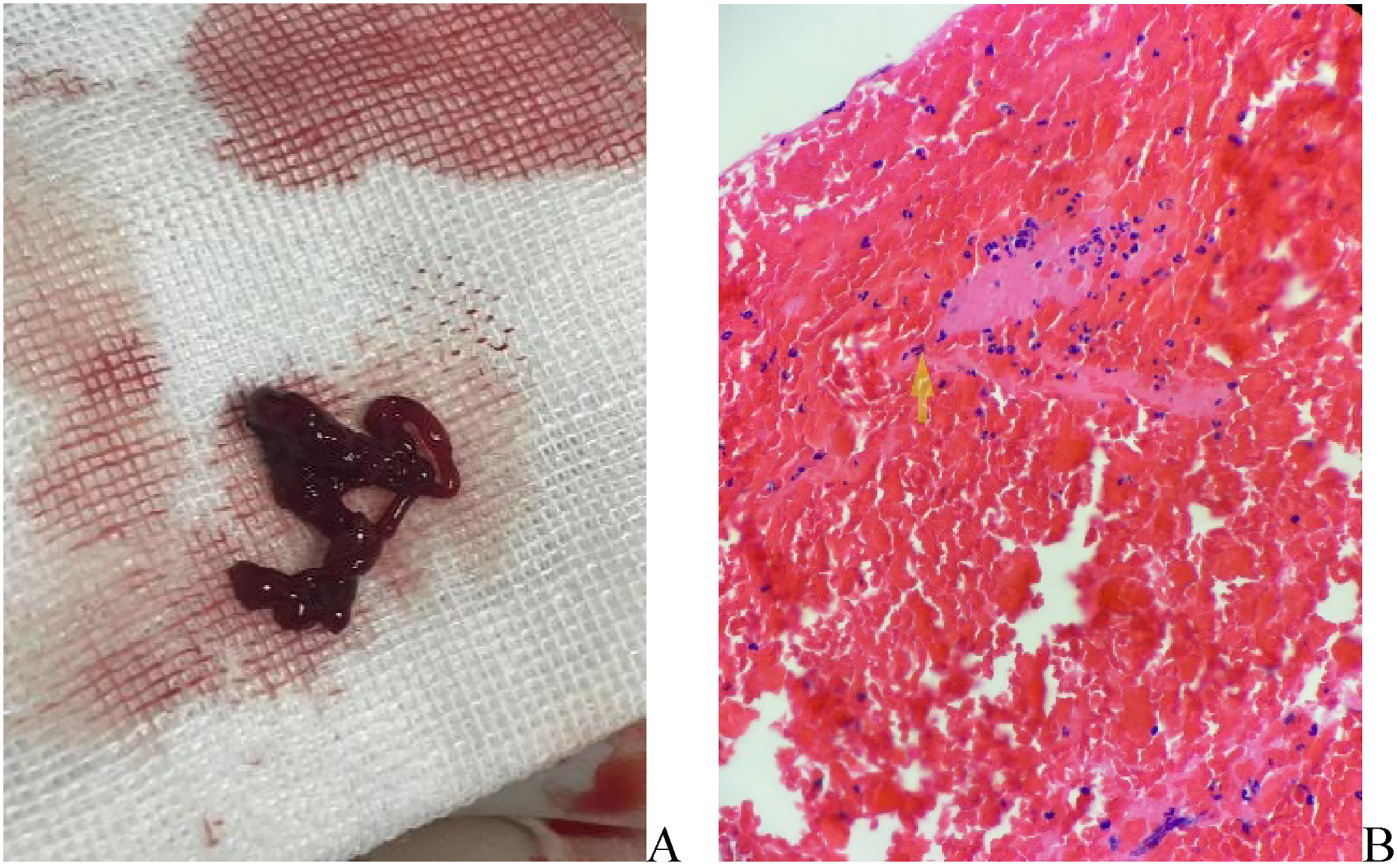
**(A)** Artery thrombosis in the brain, gray -brown strip tissue, a volume of about 1 cubic centimeter; **(B)** mixed fibrin, a large number of red blood cells, neutrophils and scattered lymphocytes, a mixed thrombus.

To further investigate the cause, transthoracic echocardiography (TTE) on the second day after admission revealed an irregular echo mass attached to the interatrial septum at the root of the anterior mitral valve. It was approximately 32 mm × 34 mm and of high activity, existing in the left atrium, mitral orifice, and left ventricle during diastole, resulting in severe mitral stenosis (about half into the left ventricle), which then entered the left atrium during systole (Figure 1, Supplementary Video S1). Left atrial myxoma was highly suspected, and the patient has no family history of similar diseases. After careful assessment of the patien's condition, to prevent the potential risk of systemic embolism, we immediately administered a hypodermic injection of 100 IU/kg low molecular weight heparin (LMWH) while awaiting surgery to remove the AM. On the fifth day of admission, despite the use of LMWH, she suddenly developed right hemiplegia and aphasia, with a National Institute of Health stroke scale (NIHSS) score of 14 and a Glasgow Coma Scale of 8. CT angiography did not show the left middle cerebral artery (MCA) M2 and distal, suggesting embolization with ischemic infarction in the left cerebral hemisphere (core infarction 34.5%). Therefore, a mechanical thrombectomy was performed, and intraoperative angiography revealed occlusion in the left MCA and left callosomarginal artery but no aneurysm, arteriovenous short circuit, or other abnormalities. Finally, a red or dark brown transparent thrombus measuring approximately 3.5 cm × 1 cm was successfully removed. It was confirmed as a mixed thrombus through histological examination of myxoma cells, found in the microscopic examination (Figure 2). The surgery achieved Grade TICI IIb recanalization of the artery (Supplementary Figure S2). The patient was transferred to the intensive care unit after surgery. Considering that there were no changes suggestive of atherosclerosis or embolus resulting from the shedding of thrombus-attached myxoma, the application of antiplatelet drugs may not have a curative effect, so we temporarily prescribed edaravone, mannitol, and symptomatic treatment for her lung infection and cardiac insufficiency symptoms.

**Figure 3:**
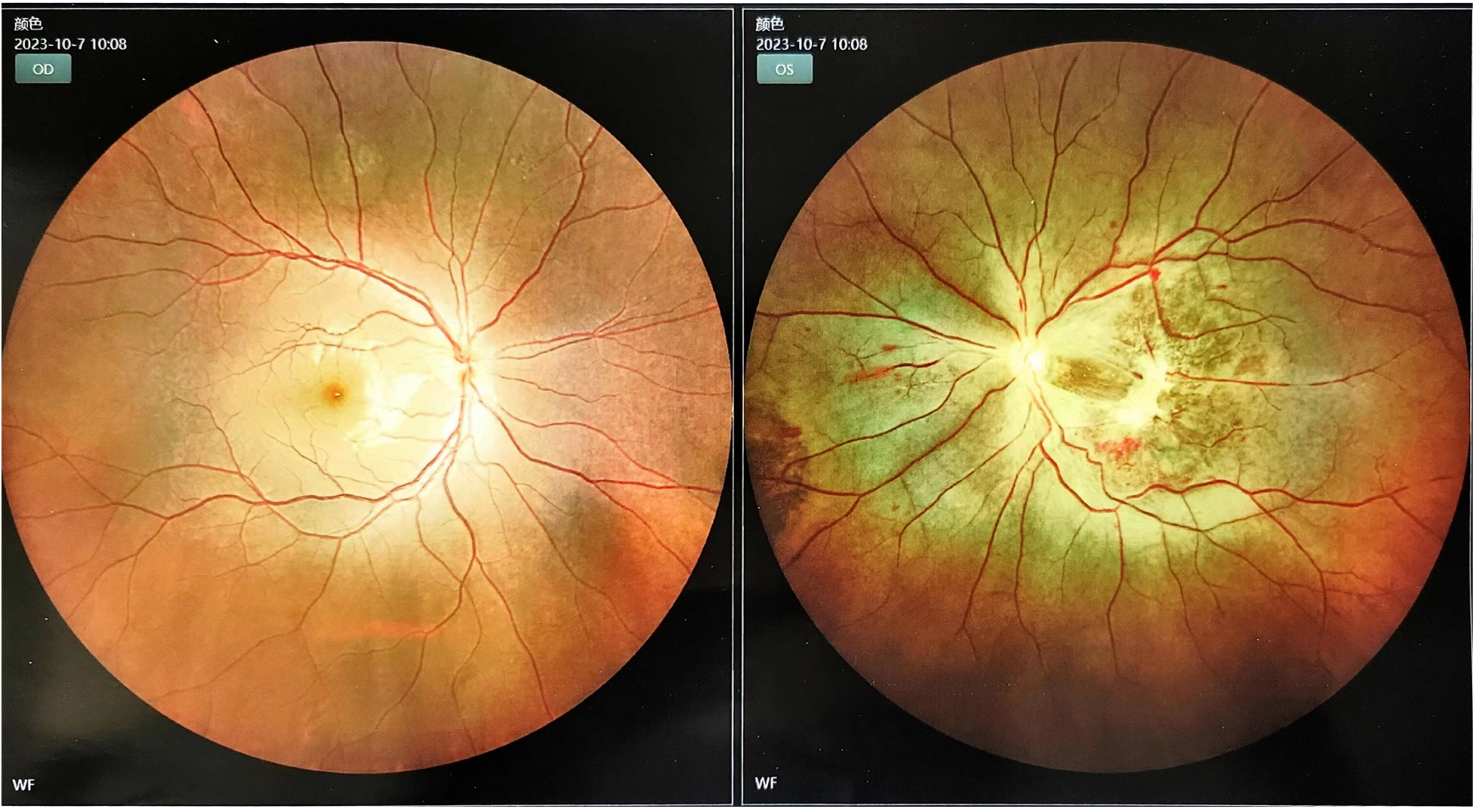
Left retinal edema due to occlusion of the central retinal artery.

Considering that the patient had a large cerebral infarction and severe mitral stenosis, we had a multidisciplinary discussion that the heparinization required for open-heart surgery may further aggravate the bleeding tendency, leading to a hemorrhagic transformation of the infarct lesion. However, as the mitral valve obstruction worsened, emergency resection of the AM was performed on the fifth day after mechanical thrombectomy. While awaiting the operation, we discontinued the LWMH and excised a pedicled dark red mass near the root of the mitral valve, which was approximately 5.5 × 4.5 × 3 cm. It was confirmed as an AM (Supplementary Figure S3). Postoperatively, no new stroke, hemorrhagic transformation of the infarct, or peripheral vascular embolism were observed. Notably, post-stroke, the physical examination revealed an enlargement of the left pupil with a diameter of approximately 4 mm, with a loss of the light sensation. After the two operations, owing to a consciousness disorder and aphasia, the patient could not accurately express symptoms of vision loss in the left eye. When the symptoms improved, fundus photography confirmed an embolism in the left central retinal artery (Figure 3). On the fourth day after resection of the AM, she was treated with a subcutaneous injection of LMWH, bridging to oral rivaroxaban anticoagulation, and no further embolic events occurred after that. The patient was discharged on the 31st day with an NIHSS score of 7.

During a 3-month follow-up, no recurrence of myxoma was found, no cerebrovascular events occurred, and rehabilitation treatment was performed regularly. The patient still had central facial paralysis, no light sensation in the left eye, right upper limb muscle strength of grade 2/5, right lower limb muscle strength of grade 4/5, limited speech, and a Modified Rankin Scale (mRS) score of 3.

Informed consent was obtained from the patient for publication of this case report. This study was performed in accordance with the Helsinki Declaration of 1964 and its later amendments. This study was approved by the Medical Review Committee of the Affiliated Hospital of Qinghai University. (SL-202453).

## Discussion

Cardiac myxoma (CM) is the most common benign tumor of the heart, with approximately 75%–85% of myxomas located in the left atrium, 10%–20% located in the right atrium, and 5% located in the ventricle (6); however, the mechanism of myxoma formation is still unknown. It has a potential risk of erosion and embolism and a similar polyp or papillary appearance, with papillary myxoma more likely leading to embolization (7, 8), resulting in complex clinical symptoms and various onset forms of embolic events. Upper and lower extremity ischemia, pulmonary embolism, mesenteric ischemia with acute abdomen, and acute coronary syndrome are the most common clinical syndromes, which occur in patients with CM due to embolization (9). Also, cerebrovascular, peripheral vascular, and central retinal artery embolization can occur (8). The cerebrovascular events may cause visual disorder, headache, atony of limbs, and epilepsy (10), which affect patients’ quality of life. However, pulmonary embolization and acute coronary syndrome may lead to a life-threatening emergency; Notably, the tumor embolus is not related to the size of the myxoma but to the mobility and fragility of the mass; the proximity of the myxoma to the mitral valve orifice causes diastolic mitral obstruction and regurgitation, thus leading to exertional dyspnea, palpitations, chest pain, and decreased activity endurance in patients. Some of them may have the characteristic “tumor plop” after S2 during heart examination (11). Particularly, large and highly mobile myxomas, such as our patient, can enter the left ventricle through the mitral valve orifice, which is a high risk of sudden death, and surgical resection is an effective treatment (9). Additionally, patients with AM often have unexplained fever, weight loss, fatigue, arthralgia, or myalgia with accelerated ESR and elevated levels of inflammatory markers, such as CRP; Currently, it is thought that these inflammatory changes are caused by myxoma through the induction of interleukin-6, but the exact mechanism is unclear (12); In our case, in addition to the recurrent chest pain and palpitations, no specific signs were found, and the first symptoms during medical consultation were dizziness, nausea, and vomiting; therefore, it was difficult to locate the site accurately. Although cranial CT detected softening foci in the temporal lobe, there were no abnormalities in the vertebrobasilar artery feeding area. Laboratory tests on admission showed rapid ESR and elevated CRP, as well as the abnormal findings of myocardial markers, which could not clearly explain the sudden dizziness, vomiting, and recurrent chest pain and palpitations, nor could they explain the presence of suspicious softening lesions in the temporal lobe without any factors or related medical history. This caused great difficulty in the initial diagnosis. Previously, a patient with AM and dizziness due to posterior circulation infarction was reported (13); benign vertigo has also been reported but is very rare (14). TTE is valuable for the early diagnosis of AM (15, 16). In our patient, TTE revealed that the myxoma swung with the mitral valve during the cardiac cycle, causing obstruction of the mitral valve, corresponding to the patien's symptoms. However, owing to her atypical symptoms and signs, our patient failed to complete TTE immediately on the day of admission, combined with waiting time for TTE. This delayed the identification and diagnosis of AM as well as the timing of myxoma surgery. Moreover, physical examination after the stroke revealed that enlargement of the left pupil and disappearance of the light reflex were signs of a left central retinal artery embolism. Therefore, for patients with a high suspicion of AM, we need to closely observe the pupil and other possible systemic embolism symptoms for timely intervention and treatment.

Currently, there are no clear guidelines for the treatment of myxomas associated with acute ischemic stroke. Previous studies have shown that 9%–22% of AM patients have cerebral embolism (17, 18). For patients with myxoma, the safety of thrombolytic therapy has been reported previously (19–22), especially for cerebral embolism caused by the loss of a tumor-attached thrombus; nevertheless, the effect of embolization of tumor fragments is poor. However, due to the fragility of myxoma, thrombolytic embolus fragments often spread to distant areas, and bleeding after thrombolysis has also been reported (23); The prognosis of patients is usually poor, and it also causes difficulties in the subsequent resection and management of myxoma. The American Heart Association guidelines weakly recommend intravenous thrombolysis with rt-PA in acute ischemic stroke due to AM, and the European guidelines recommend intravenous thrombolysis with rt-PA in patients with unruptured aneurysms less than 4.5 h, both of which have a low level of evidence (24, 25).

Meanwhile, it has been reported that intracranial myxoma aneurysm is detected in patients with AM after repeated stroke in consultation (26). Therefore, the risk of intracranial bleeding in patients with high suspicion of myxoma must be evaluated. Digital subtraction angiography (DSA) is a necessary diagnostic method, especially for patients who are temporarily unable to complete myxoma resection surgery. Currently, there are reports of using tirofiban for stroke in patients with myxoma, showing a good prognosis. However, distal dissemination of the embolus cannot be avoided, and its efficacy and safety still need to be further verified (27).

Concurrently, the curative effect of mechanical thrombolysis for patients with stroke caused by AM is certain, especially for those with intracranial vascular proximal occlusion. Mechanical thrombolysis has shown good results for prognosis and recanalization, as reported in both pediatric and adult patients with AM (28–30). Consequently, our patient underwent a mechanical thrombectomy immediately after the stroke.

Thus, the most effective treatment is resection of the AM, which is safe and has a low mortality rate of less than 5%. However, there are no definite guidelines for a specific safe time interval from the onset of stroke to surgical resection. Although previous studies have shown that direct heart surgery is safe for patients with acute cerebral infarction (31), in patients with stroke, the bleeding risk after heparinization should be carefully evaluated, including the general condition, risk of other related complications, and whether the patient can tolerate the operation. Some researchers believe that bridging therapy can be performed with anticoagulation therapy to delay CM resection (17), but delayed surgical removal of AM may be associated with an increased risk of complications (32). In our case, the embolus causing middle cerebral artery occlusion was mixed with a thrombus rather than a tumor embolus. LMWH anticoagulation before the resection of the myxoma failed to successfully prevent cerebrovascular events. The source of the thrombus embolus was believed to be the shedding of the myxoma attached to the thrombus. LMWH has a fibrinolytic-promoting effect, therefore, we investigated whether stroke due to instability and detachment of the attached thrombus is associated with the use of LMWH. A retrospective study shows that about 23% of patients have embolization despite the use of anticoagulation therapy (33). LMWH did not prevent stroke in our patients, even with the aggravation of intracardiac obstruction symptoms after acute cerebral infarction being life-threatening. Emergency resection of myxoma after mechanical thrombectomy has also been achieved with a good prognosis. Therefore, individualized multidisciplinary assessments are necessary. Hence, what is the role of anticoagulants in the perioperative period of AM resection? For patients with previous embolic events, we aimed to determine whether perioperative anticoagulation during AM resection is effective. We searched the PubMed database and reviewed relevant case reports, which are listed in Table 2.

**Table 2:** Published cases of perioperative anticoagulation therapy after embolism of atrial myxoma.

Reference	Age/sex	Symptom	Location	Therapy before cardiac surgery	Embolism before cardiac surgery	Outcome
Waikar et al. (34)	23/F	Loss of consciousness, convulsion	Left MCA territory	LMWH, enoxaparin; 1 day before surgery, change to aspirin	Non	Aphasia at 1 year
Shrestha et al. (35)	55/M	Headache, vomiting, double vision, unsteady gait	PICA territory	Anticoagulants	Splenic infarct, bilateral renal infarct	Well at 4 month
Sadia et al. (36)	44/F	Right-sided weakness, unintentional weight loss	Left MCA and PCA territory	Unfractionated heparin, atorvastatin	Non	Neurological manifestations improved at 3 month
Esmaeili et al. (21)	31/M	Left hemiparesis	Right MCA territory	After intravenous thrombolysis, aspirin 80 mg/day, clopidogrel 75 mg/day, and heparin, on 4 days before surgery change to heparin 900 U/h	Non	Discharge with NIHSS 0
Gil et al. (37)	61/F	Vertigo, left hemiparesis	Multiple foci of infarction in the frontal, parietal, and occipital cortex.	Anticoagulation	Non	Well
Berrada et al. (38)	75/M	Angina pectoris, right hemiparesis	Left hemisphere acute and old luminal infarction	Aspirin, clopidogrel, enoxaparin	Non	Non
Saaf et al. (39)	55/F	Left hemiparesis, mild paralytic dysarthria, facial central paralysis	The pons and the anterior bulbar area	Enoxaparin 4,000 IU associated with clopidogrel; 5 days before surgery change to enoxaparin 4,000 IU	Non	Well
Mackie et al. (40)	39/F	Unresponsive and in respiratory distress	Left MCA territory	Aspirin (325 mg), and an intravenous heparin drip	Iliac artery, renal artery, left MCA, and epicardial branches of the left main coronary artery	Death
Kohno et al. (41)	79/M	Altered consciousness	Left MCA territory	Intravenous thrombolysis, and warfarin 14 days after stroke onset with PT-INR: 2.0–3.0	Non	The right hemiparesis and aphasia did not improve. mRS 4 on day 36
Abe et al. (42)	70/M	Aphasia, right facial paralysis, right hemiparesis, and sensory disturbance	Left MCA territory	Alteplase (0.6 mg/kg), warfarin	Non	mRS 0
Our case	27/F	Dizziness and palpitations	Left MCA territory	LMWH	Left MCA territory, left central retinal artery occlusion	mRS 4 at 3 month

F, female; M, male; MCA, middle cerebral artery; PCA, posterior cerebral artery; PICA, posterior inferior cerebellar artery; LMWH, low molecular weight heparin; NIHSS, National Institute of Health stroke scale; mRS, modified rankin scale.

In Table 2, all the cases were patients with previous or recent cerebrovascular events and received anticoagulation therapy after cerebrovascular events; 27% of patients, including our patient, developed arterial embolism, including renal artery, splenic artery, coronary artery, central cerebral artery and central retinal artery. Notably, in the literature review, embolism of the central retinal artery has not been frequently reported, and it is difficult to detect by DSA examination. Additionally, when the patient has poor consciousness or aphasia and cannot express the symptoms of vision loss, this specific embolic symptom is often ignored, so it is necessary to examine the pupil and fundus. In the cases reported by Benjamin et al., although 325 mg of aspirin and intravenous heparin were used after acute coronary syndrome, embolization was not prevented, and the embolus was proven to result from the dissemination of myxoma fragments (38). Our patient had a mixed thrombotic embolus, and other authors did not report histological data on the embolus. Most patients were unaware of the presence of AM in advance, and the symptoms were mostly consistent with hemiplegia and aphasia caused by stroke. In the initial treatment plan, owing to the high possibility of cardiac embolism, patients received anticoagulation therapy, with some even using dual antiplatelet drugs simultaneously, and no embolization occurred during the waiting period for surgery. For patients within the time window, anticoagulation was also administered in a timely manner after intravenous thrombolysis, resulting in no perioperative embolism and a good clinical prognosis. Given the potential embolic risk, especially the high suspicion of embolism resulting from the shedding of an attached thrombus, anticoagulation therapy for the prevention of embolism should be considered. Embolism caused by tumor embolus still needs to be considered, which often suggests a worse response to thrombolysis, anticoagulant curative effect, and prognosis. However, the efficacy and prognosis of anticoagulation for the prevention of embolism in patients with AM need further experimental verification. The most critical treatment is surgical resection, and in the majority of cases reviewed, the patients recovered well after surgery and showed no related complications.

For this patient, the treatment was successful. Additionally, it should be mentioned that this case of AM had a previous embolism history, and it is not possible to trace the time of previous embolic symptoms. Theoretically, stroke recurrence, cannot completely exclude factors such as myxoma swing and hemodynamic instability. Anticoagulant efficacy in AM-associated AIS needs to be assessed in the future. Also, more extensive multicenter studies are needed to understand better the risk factors and mechanisms of embolization in AM patients to improve diagnostic accuracy and therapeutic approaches.

## Conclusion

In conclusion, we reported a case of AM with suspected intracranial softening lesions and sudden dizziness symptoms, in which the patient suffered from a large cerebral infarction caused by thromboembolism after anticoagulation and was successfully treated by AM resection in the acute stage of cerebral infarction after mechanical thrombectomy.

Therefore, for these patients TTE should be completed as soon as possible, and the waiting time for myxoma resection should be minimized. Although there are no clear guidelines recommended, we suggest perioperative anticoagulation treatment for patients with myxoma-related stroke undergoing thrombolysis, those with a low risk of bleeding after mechanical thrombectomy, and those who have not yet developed an embolism. This may help prevent the potential systemic embolism, with surgical resection being the most effective treatment.

## Data Availability

The raw data supporting the conclusions of this article will be made available by the authors, without undue reservation.
