# Supplementary material for: Preoperative management and anticoagulant efficacy in atrial myxoma-associated acute ischemic stroke: a case report and literature review
Source: Front Cardiovasc Med. 2024 Nov 12;11:1435047. doi: 10.3389/fcvm.2024.1435047 (PMC11588714; doi:10.3389/fcvm.2024.1435047)
Supplement: Supplementary file 1 [file Datasheet1.docx]

# Supplementary files

**Figures**


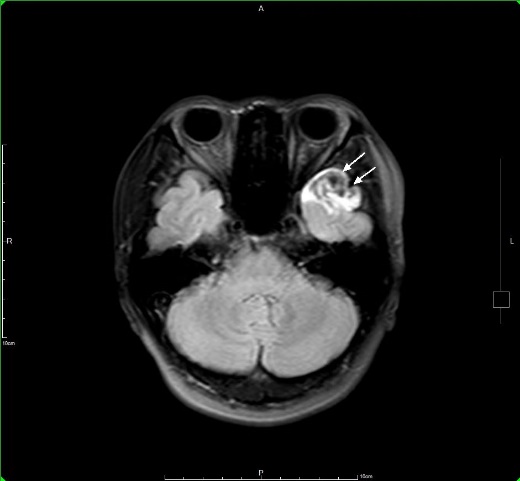

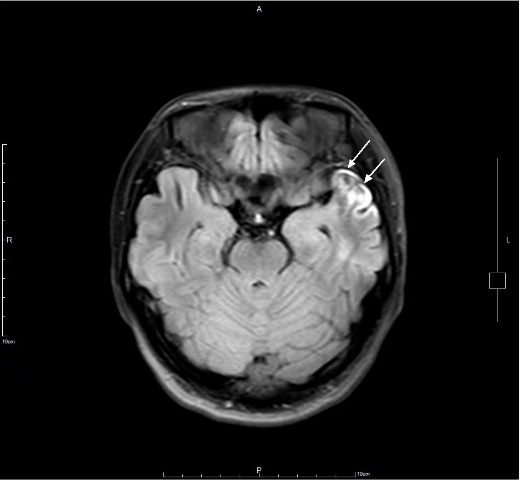


Figure 1 FLAIR in the left temporal lobe showed a promiscuous signal, considering a line-softening focus with gliosis


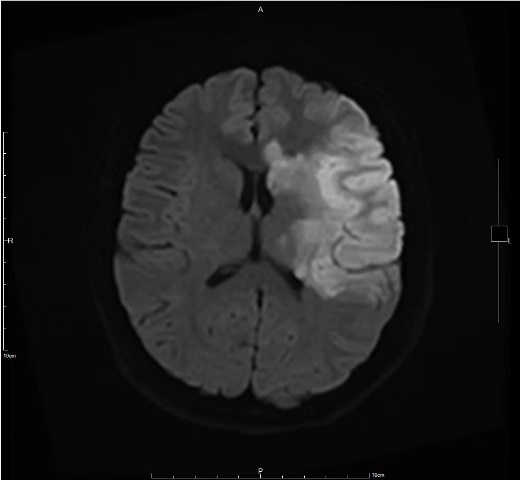
 A
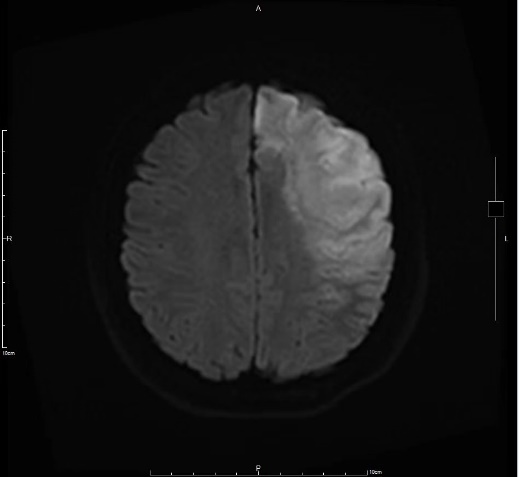
 B


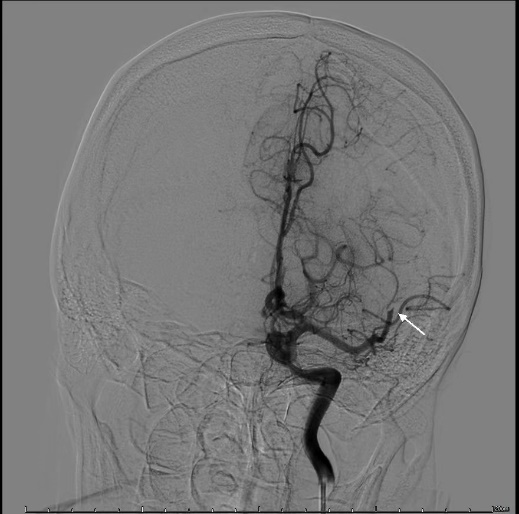
 C
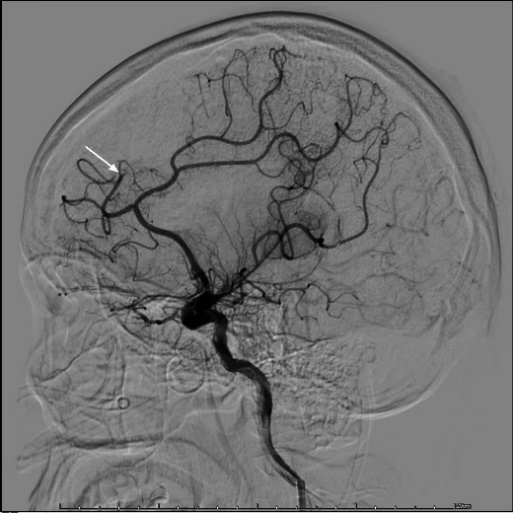
 D


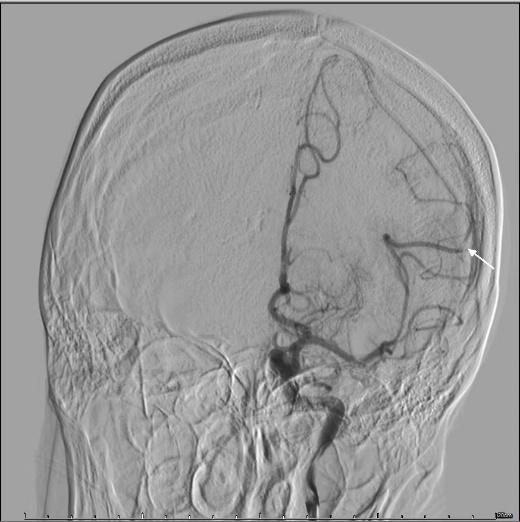
 E

Figure 2

A, B Large acute phase of infarction in left frontal, temporal, and parietal cerebral;

C, D Occlusion in the left MCA and left callosomarginal artery;

E After recanalization, the M4 segment of artery in the left MCA was embolized;


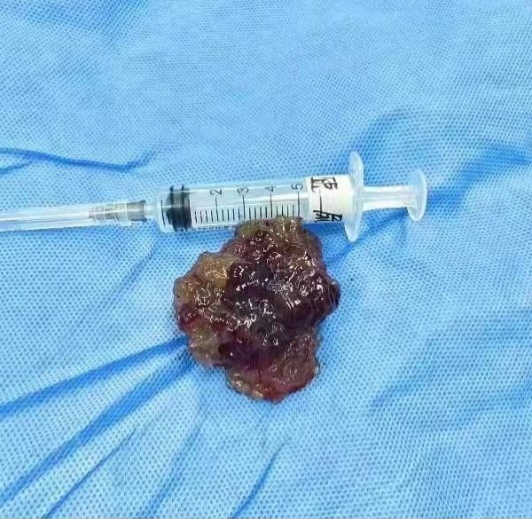
 A
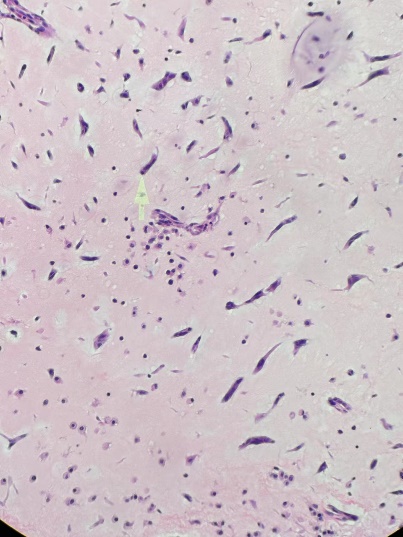
 B


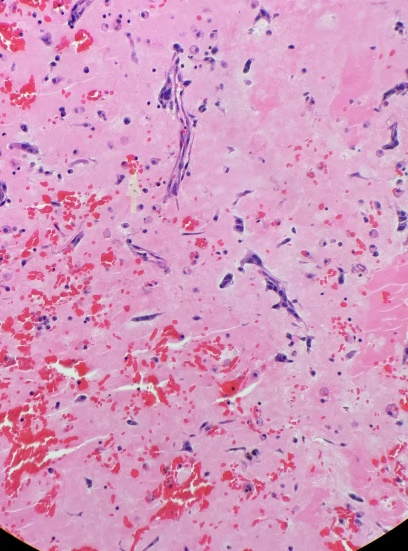
 C

Figure 3

A Atrial myxoma, a grayish-white brown soft tissue with a volume of about 5.5cm*4.5cm * 3cm, showed a polypapillary surface and gray parenchyma;

B, C In a mucus background, spindle cells are visible, as well as star-awn tumor cells and red blood cells;

Video 1 Swing of an atrial myxoma between the left atrium and the left ventricle

**Timeline**

TTE revealed an irregular echo mass attached to the interatrial septum at the root of the anterior mitral valve.

ESR 47 mm/h, CRP 4.82 mg/L, creatine kinase isoenzyme 38U/L, and myoglobin, 166 ng/mL.

Extreme low-density imaging was observed in the left temporal region of the patient’s head CT scan

The mechanical thrombectomy was performed, and intraoperative angiography revealed occlusion in the left MCA and left callosomarginal artery.

Right hemiplegia and aphasia with an NIHSS score of 14, and the left pupil was enlarged

Discharged on the 31st day

Past 5 months

The fundus photography confirmed an embolism in the left central retinal artery artery.

Admission

Day 2

Day 5

Day 9

Day 30

The myxoma was removed

Palpitations, chest pain, and fatigue

Administered a hypodermic injection of 100 IU/kg LMWH

Dizziness with visual rotation for 1 day
